# Our Journal Next Year

**Published:** 1880-12-20

**Authors:** 


### *OUR JOURNAL NEXT YEAR.*

Evidences of the present and growing popularity of the *Southern Medical Record* are manifest in the renewals of subscription almost daily coming in, and the frequent additions to our list of new names from various sections of the country. The renewals very frequently make favorable mention of our Journal—its practical features and the satisfaction which they derive from perusing its terse and well selected pages. These facts are very satisfactory to the Editors, and will encourage them to renewed and stronger efforts to increase its interest and usefulness to our readers.

